# Challenges and opportunities in vascularized composite allotransplantation of joints: a systematic literature review

**DOI:** 10.3389/fimmu.2023.1179195

**Published:** 2023-05-19

**Authors:** Lei Zhang, Isabel Arenas Hoyos, Cédric Zubler, Robert Rieben, Mihai Constantinescu, Radu Olariu

**Affiliations:** ^1^ Department of Plastic and Hand Surgery, Inselspital University Hospital Bern, University of Bern, Bern, Switzerland; ^2^ Department for BioMedical Research, University of Bern, Bern, Switzerland; ^3^ Department of Plastic and Reconstructive Surgery, Plastic and Reconstructive Surgery Center, Zhejiang Provincial People’s Hospital, Hangzhou, China

**Keywords:** joint allotransplantation, knee allotransplantation, elbow allotransplantation, vascularized composite allotransplantation, functional reconstruction

## Abstract

**Background:**

Joint allotransplantation (JA) within the field of vascularized composite allotransplantation (VCA) holds great potential for functional and non-prosthetic reconstruction of severely damaged joints. However, clinical use of JA remains limited due to the immune rejection associated with all forms of allotransplantation. In this study, we aim to provide a comprehensive overview of the current state of JA through a systematic review of clinical, animal, and immunological studies on this topic.

**Methods:**

We conducted a systematic literature review in accordance with the PRISMA guidelines to identify relevant articles in PubMed, Cochrane Library, and Web of Science databases. The results were analyzed, and potential future prospects were discussed in detail.

**Results:**

Our review included 14 articles describing relevant developments in JA. Currently, most JA-related research is being performed in small animal models, demonstrating graft survival and functional restoration with short-term immunosuppression. In human patients, only six knee allotransplantations have been performed to date, with all grafts ultimately failing and a maximum graft survival of 56 months.

**Conclusion:**

Research on joint allotransplantation has been limited over the last 20 years due to the rarity of clinical applications, the complex nature of surgical procedures, and uncertain outcomes stemming from immune rejection. However, the key to overcoming these challenges lies in extending graft survival and minimizing immunosuppressive side effects. With the emergence of new immunosuppressive strategies, the feasibility and clinical potential of vascularized joint allotransplantation warrants further investigation.

## Introduction

Vascularized composite allotransplantation (VCA) is a relatively new field that offers functional restoration of severely damaged limbs. Joint allotransplantation (JA), a specialized subgroup within VCA, aims to replace joints such as the elbow or knee (1–4). Compared to other VCA procedures, JA provides a segmental transplantation that can potentially offer significant functional restoration of the affected joint (5–8). Although limited clinical experience suggests that knee transplantation should only be considered as a last resort for patients with extensive loss of cartilage and bone, deficient extensor mechanism, and soft tissue and skin defects, JA has the advantage of offering functional reconstruction in cases where modern prostheses have failed (9–13).

However, as with other VCA procedures, immune rejection is the main obstacle to wider clinical application of JA. Studies have shown that, while technically and anatomically feasible, long-term survival of allografts has been limited due to serious immune rejection (2, 3, 14, 15). In addition, the necessary life-long immunosuppression carries risks such as increased susceptibility to infection and malignancy, which need to be balanced against the benefits of functional joint reconstruction (16, 17).

Research and clinical application of JA have stagnated recently, potentially due to these considerations. In this study, we aim to systematically review the current literature on joint-specific allotransplantations, summarizing relevant clinical studies, animal models, immune processes, rejection, and functional aspects involved. We will also discuss the prospects of novel immunosuppressive strategies, as well as future challenges in JA.

## Methods

**Figure 1:**
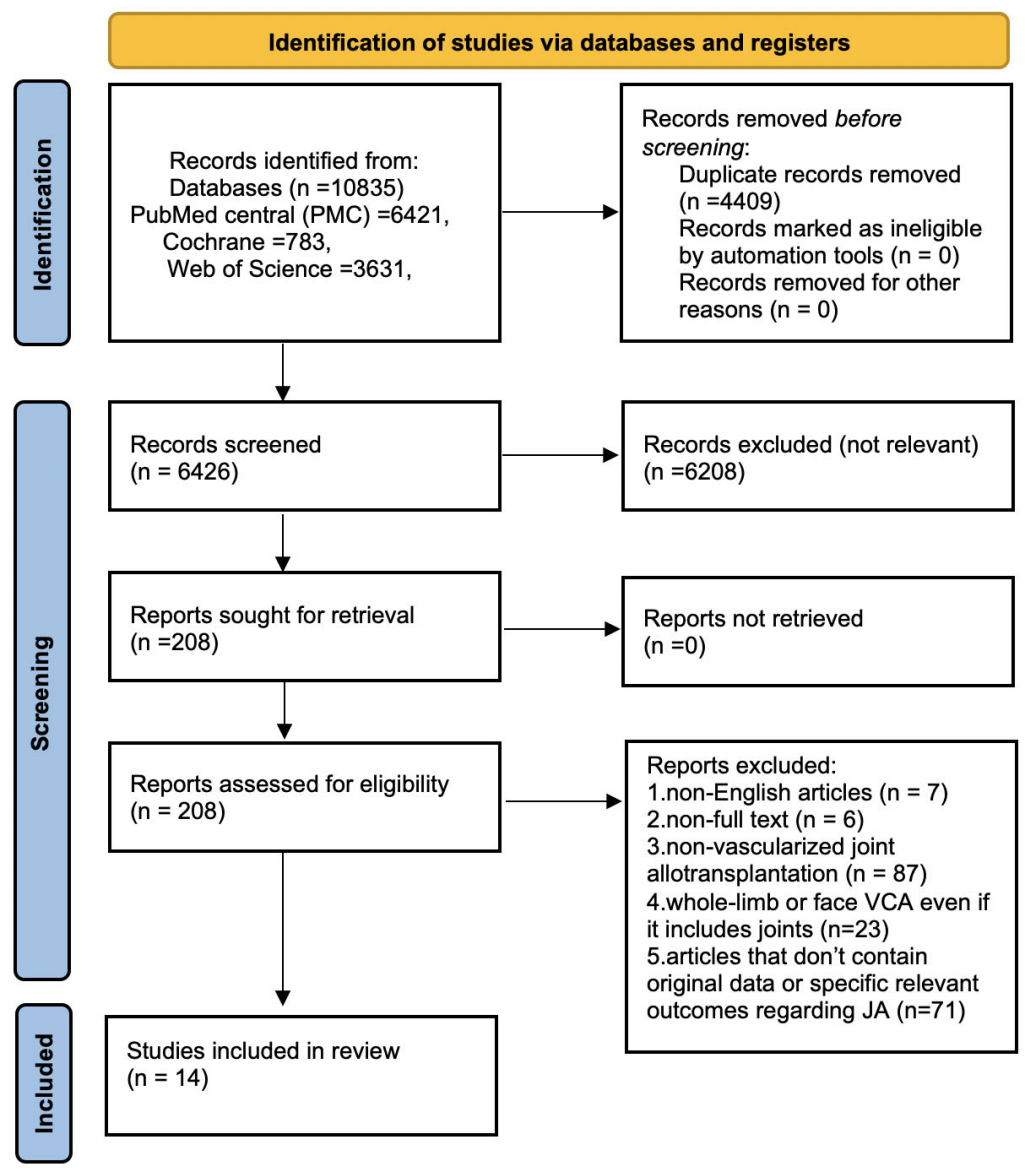
Search strategy flow chart in accordance with the PRISMA 2020 guidelines for eligible articles.

We conducted a systematic literature review following the PRISMA guidelines (Preferred Reporting Items for Systematic Reviews and Meta-Analyses, PRISMA 2020) (18) using the search terms (((joint) AND (allotransplantation)) OR ((joint) AND (allograft)) OR ((knee) AND (allotransplantation)) OR ((elbow) AND (allotransplantation))) in the PubMed, Cochrane Library, and Web of Science databases. We included articles on clinical experience, anatomical studies, and joint allotransplantation-specific animal experiments, and applied the following exclusion criteria: (1) non-English articles, (2) inaccessible full text, (3) non-vascularized joint allotransplantation, (4) whole-limb or face VCA (vascularized composite allotransplantation), even if it includes joints, and (5) articles that do not contain original data or specific relevant outcomes regarding joint allotransplantation. **Figure 1** shows the flow chart detailing the search strategy and inclusion of eligible articles.

## Results

Based on the inclusion and exclusion criteria, we included 14 studies in this review. Seven of them represent joint allotransplantation-specific papers on experimental animal research (**Table 1**), while the other seven studies included three articles reporting clinical cases, three anatomical cadaver studies, and one CT-scan-based article (**Table 2**).

**Table 1:** Overview of animal experiments in joint allotransplantation.

Author and year	Species	Model	Immunosuppression regimen	Postoperative graft evaluation	Major results and conclusions	References
Rosso et al. 1997	Dogs	Knee allotransplantation	Cyclosporin A for 1 week before the transplantation	1. Graft vascularization 2. Fluorography 3. Weight bearing	Proof of principle: joint allotransplantation is possible and critically dependent on rejection control.	(19)
Vögelin E et al. 2002	Rats	Knee allotransplantation	1. Rapamycin 2. Mycophenolate Mofetil 3. Tacrolimus	(-)	Long-term intermittent immunosuppression with tacrolimus was significantly superior to rapamycin and mycophenolate mofetil in preventing rejection of the transplanted articular cartilage of a vascularized knee joint allograft up to 1 year after surgery.	(20)
Larsen et al. 2010	Rats	Elbow allotransplantation	Tacrolimus administered daily	1. Pedicle patency 2. Blood flow in the bone 3. Capillary sprouting	Development of a vascularized elbow allotransplantation model in the rat.	(21)
Kremer et al. 2012, 2013	Rabbits	Knee allotransplantation with superficial inferior epigastric fascial flap and arteriovenous bundle	Short term (2 weeks or 3 weeks) period of postoperative tacrolimus	1. Radiography 2. Microangiography 3. Biomechanical tests	Surgical angiogenesis from implanted autologous tissue with short term IS improves bone viability, healing, and material properties.	(22, 23)
Shibuya et al. 2014	Rats	Knee allotransplantation	(-)	(-)	Chondrocytes undergoing apoptosis in allotransplantation may induce acute rejection.	(24)
Tang et al. 2015	Rats	Elbow allotransplantation	1. Group1: Cyclosporin A for long-term 2. Group2: Cyclosporin A for 10 days 3. No Immunosuppression	1. Pedicle patency 2. Graft blood flow, bone union 3. Joint architecture	Animals with ongoing IS regained function and maintained grossly normal elbow cartilage compared to severe rejection without IS or with only short-term immunosuppression	(25)

**Table 2:** Overview of clinical and cadaver studies of joint allotransplantation.

Author and year	Object	Operation	Immunosuppression regimen	Functional evaluation	Major results and conclusions	References
Hofmann research team. 2000, 2007, 2011	Patients	Knee allotransplantations	Antithymocyte Globulin, Cyclosporin A, Azathioprine and Methylprednisolone Tacrolimus	1. Radiography 2. Range of motion	Full weight-bearing and range of motion were achieved. All knee grafts were lost within 56 months.	(2–4)
Steinberger et al. 2017	Cadavers	Exploration of elbow anatomy and cadaveric elbow JA	(-)	(-)	Proof of principle: Elbow JA is technically and anatomically feasible.	(14)
Khavanin et al. 2018	CT scans and cadavers	Maxillofacial CT scans of mandibular morphology were analyzed, and temporomandibular joint harvest and transplantations were performed in cadavers	(-)	1. Radiography 2. Range of motion	Proof of principle: Allotransplantation of the mandible and bilateral condyles is technically and anatomically feasible with acceptable immediate postoperative jaw position and range of motion in the cadaver.	(9, 10)
Pet et al. 2018	Cadavers	Exploration of elbow anatomy and cadaveric elbow JA	(-)	(-)	A vascularized elbow joint design and surgical technique for graft harvest are proposed.	(15)

The animal studies on JA mainly used small animal models in rats and rabbits, with just one proof of principle study in dogs in the 90s. The studies employed different immunosuppression regimes for different durations, which were compared. **Table 1** provides a description of the findings of the experimental studies.

Currently, knee joint allotransplantation is the only joint allotransplantation that has been performed in humans. Six cases of knee allotransplantation have been reported in the literature between 1996 and 2004. The first five cases used anti-thymocyte globulin as an induction therapy, along with cyclosporin A, azathioprine, and methylprednisolone as immunosuppressive regimens. However, these five knee allografts were lost within three years. One graft was lost due to surgical site infection, while the other four grafts suffered graft rejection at different stages, including one due to noncompliance (4). The sixth patient received tacrolimus and mycophenolate mofetil and had an acceptable range of motion of the transplanted knee. He was ambulatory and full weight-bearing during long-term surveillance. However, at 50 months, knee function decreased from 0-0-90 degrees flexion to 0-10-40 degrees, and anterior instability developed. The knee graft was eventually lost due to late rejection vasculopathy at 56 months following transplantation (4).

In addition to clinical studies, anatomical human cadaveric studies focusing on the technical feasibility of elbow and temporomandibular joint allotransplantation have shown that both are technically and anatomically feasible. **Table 2** summarizes the findings of these studies.

## Discussion

### Experimental animal models

Animal models have been used to investigate JA, with small animals such as rats and rabbits being the primary models (**Table 1**). In rat models of elbow JA, successful outcomes require anastomosis of the brachial artery and median nerve neurorrhaphy, internal fixation of the joint, and soft tissue coverage with recipient musculature (20, 21, 24, 25). Similarly, knee allotransplantations in rabbits have been found to provide good skeletal stability during long-term observation (26). To improve graft viability without the need for long-term immunosuppression, Kremer et al. introduced surgical neoangiogenesis in rabbit knee allotransplantations using a superficial inferior epigastric fascial flap and a saphenous arteriovenous bundle to generate a neoangiogenic bone circulation (22, 23).

Despite their advantages, small animal models have limitations when compared to humans and large animals. These include differences in genome, immunological behavior, and biomechanical parameters such as weight load and range of motion (27–29). Hence, small animal models are typically used as the first step in any *in vivo* research. For example, chimerism has been induced in small animal models to overcome immunological barriers in VCA, but this has not been achieved in larger animals or humans (30, 31).

Large animal models can serve as an intermediate step before clinical application in humans. However, joint-specific allotransplantations have not been widely reported in this setting. Knee allotransplantations in dogs were reported over 20 years ago, but these were proof-of-principle reports lacking surgical details and radiological follow-up (19, 32). The focus then shifted towards heterotopic swine hind limb and porcine orthotopic forelimb models, which are currently widely used in VCA research (33–35). It appears to be more challenging to establish joint-specific large animal models for JA compared to whole limb allotransplantations. JA requires the precise dissection of structures surrounding the joint, such as ligaments, muscles, vessels, and regional nerves, in both the donor and recipient. For orthotopic replantation, internal fixation and joint remodeling are necessary, making it more demanding than whole limb allotransplantation, which only requires bone fixation and anastomosis on one end. In addition, ensuring long-term joint stability and eliminating potential external factors during follow-up observation is arguably more challenging in large animals than in small animals due to differences in biomechanical burden. Thus, future studies are needed to establish large animal models of JA with reliable and reproducible results.

### Clinical and cadaver studies of joint allotransplantation

Several studies have demonstrated that JA in humans is technically and anatomically feasible (**Table 2**). In addition to the knee, recent investigations in human cadavers have suggested that elbow allotransplantation could also be viable based on local vascular anatomy and existing surgical techniques (14, 15). Similarly, studies of the temporomandibular joint have suggested the feasibility of JA based on geometric analysis of maxillofacial CT-scans, and subsequent transplantations in cadavers appear to confirm these findings (9–11).

In 2021, the first composite vascularized elbow autograft was reported from a left to right upper extremity. The patient was able to complete daily living activities independently 4 years postoperatively. Although an autograft was used in this case, it further illustrates the technical feasibility and potential success of elbow joint transplantations (36).

However, despite the theoretical and technical viability of JA, its reported clinical applications lag far behind those of other VCA procedures (37). Graft rejection remains the main obstacle to clinical implementation of JA, as described by several studies (2, 3, 17). The limited clinical experience available thus far has shown rather sobering results. Hofmann et al. reported a total of six knee allotransplantations, and despite the application of immunosuppressants, all patients eventually lost the grafts due to immune rejections. Ultimately, four patients underwent above-the-knee amputations and two were subject to arthrodesis (3, 4). However, before the eventual immune rejection, joint function appeared to improve gradually during long-term follow-up.

### Immune response and rejection

The survival time of knee allotransplantations is currently limited, with the longest reported survival time being 56 months. This duration is much shorter than that of whole upper limb transplantations, which have been reported to survive for more than 10 years (3, 37–39). Rejection of the transplant is more likely to occur in JA due to their specific anatomical and surgical characteristics. JA is typically performed as a segmental, partial transplantation, where both the proximal and distal ends are connected to, and almost completely surrounded by, recipient tissue. This characteristic of JA resembles solid organ transplantation rather than other VCA, and theoretically renders it more vulnerable to immune recognition and rejection (39–43). Early detection of rejection in JA is challenging since the graft is usually embedded in recipient tissue (44, 45). Therefore, the diagnosis of rejection mainly relies on patient complaints, which typically lag behind the occurrence of rejection. In previous studies, it has been suggested that inclusion of a skin flap in the JA could improve graft monitoring and immunosuppressive administration. The limited success and shorter graft survival time of JA compared to whole upper limb transplantations may be explained by a combination of these factors (4, 38).

**Figure 2:**
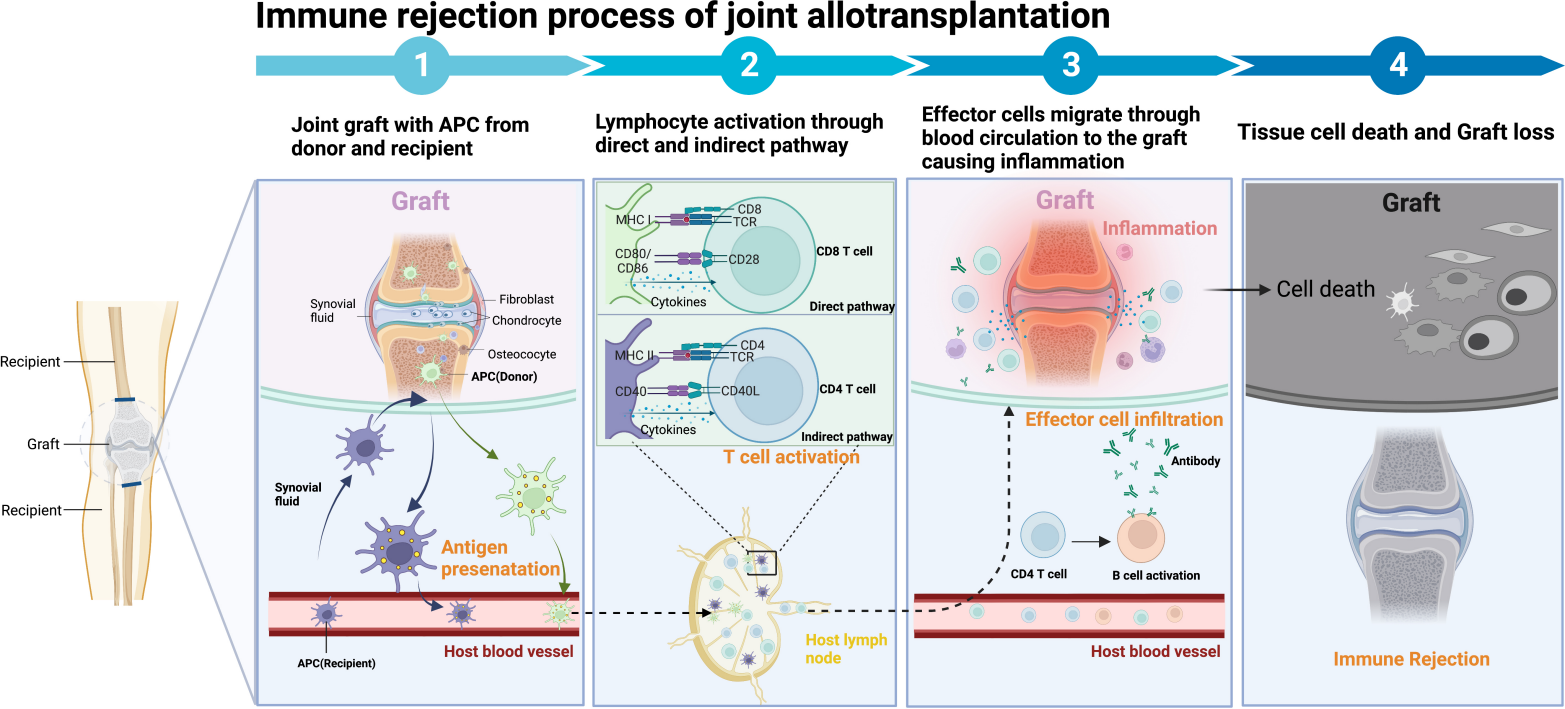
Components of joint allotransplantation and the immune response. The joint allograft comprises the articular capsule, bone, cartilage, ligaments, synovia, and possibly varying amounts of skin and muscle. Immune rejection begins with the recognition of donor major histocompatibility complex (MHC) molecules by donor antigen-presenting cells (APCs) in the direct pathway or by recipient APCs in the indirect pathway. These APCs subsequently present MHC to CD4 T helper (Th) cells, which activate immune cells such as cytotoxic T lymphocytes (CTLs), natural killer (NK) cells, macrophages, B cells, and the complement system. This immune response can induce rejection through mechanisms such as cell-mediated cytotoxicity, antibody-dependent cell-mediated cytotoxicity (ADCC), formation of the membrane attack complex (MAC), and the release of various cytokines and inflammatory mediators, eventually resulting in the death of the transplanted cells. Figure created with BioRender.com.

A joint allograft consists of multiple tissues, including articular capsule, bone, cartilage, ligament, synovia, and optionally some amount of skin and muscle. After transplantation, these tissues are targets of rejection reactions by both the innate and adaptive immune systems of the host. The initial inflammatory response – representing the ‘danger’ signal – will lead to direct and indirect graft antigen presentation – providing the ‘foreign’ signal – and finally to graft tissue damage *via* T cell-mediated cytotoxicity, antibody-dependent cell- and/or complement-mediated cytotoxicity, and other mechanisms (**Figure 2**). Mechanisms of rejection in VCA have been studied and discussed in detail, with a particular focus on the processes in the skin and soft tissues (40, 42, 46, 47). However, much less is known about the cartilage-specific rejection mechanisms, which are crucial in JA.

Bone typically represents the primary component in JA. It has been observed that the presence of vascularized bone creates a more tolerogenic environment than VCA grafts without bone (42). Previous bone VCA studies in large animal models have evaluated bone healing and remodeling and quantified bone characteristics. These studies have demonstrated that the development of a periosteal callus and new bone arising from the vascularized allotransplant led to the formation of a bridging callus, as opposed to cryopreserved allogenic bone (48). Despite these promising results, rejection of bone may still occur. Clinical studies have shown reduced or absent bone metabolism in late postoperative single photon emission computed tomography (SPECT) scans, with bone necrosis subsequently confirmed by bone biopsy in two patients with knee allotransplantations (49).

Allogeneic bone marrow transplantation offers distinct immunomodulatory benefits, enabling the formation of chimerism and even immunologic tolerance in transplantation (50, 51). This is particularly relevant for VCA, where bone marrow plays a critical role. The engraftment of the bone marrow component within the transplanted bone segment provides a native advantage in VCA, by facilitating a continuous supply of donor-derived hematopoietic progenitor cells, which in turn leads to enhanced and expedited reconstitution of transplanted tissues. Prior research has established that vascularized bone marrow plays a pivotal role in improving clinical outcomes in graft survival, as well as in promoting the formation of macrochimerism in primates (52). Furthermore, sustainable chimerism and immune tolerance have been successfully achieved in VCA mouse models *via* the engraftment of vascularized bone marrow (51). The JA approach may use the advantages of transplanted bone marrow, however the specific impact of this particular combination requires further investigation to fully realize its potential.

Cartilage serves an important functional role in JA. Studies have suggested that chondrocytes and cartilage possess immunosuppressive and immune privileged properties. Chondrocytes, for example, have been shown to express CD80/B7 inhibitors, chondromodulin I, and secrete indoleamine 2,3-dioxygenase (IDO) as well as exhibit mesenchymal lineage *in vitro*, suggesting they may be immuno-evasive (53–56). Moreover, the dense extracellular matrix (ECM) surrounding chondrocytes *in vivo* may sequester antigens and provide a physical barrier against immune detection (57–59). However, clinical studies in humans and animals do not entirely support the immune-privilege theory (58–61). Knee chondrocytes in a rat composite tissue allotransplantation model underwent apoptosis induced by acute rejection, and histopathology of human knee transplantation cases showed necrosis of articular cartilage (24). The mechanisms of cartilage rejection in JA are not well understood, although a possible explanation is that humoral signaling in the synovial fluid may play a role.

Fibrous tissues such as cruciate ligaments are typically included in knee transplantation to provide joint stability. They may, however, be attacked by the host immune system, leading to delayed remodeling and potentially impaired long-term stability compared to autografts. Clinical studies of fresh-frozen anterior cruciate ligament (ACL) allograft reconstruction have demonstrated excellent results, but also highlighted the risk of immune rejection (62–66).

In JA, skin is an optional component, but is often included due to its susceptibility to immune reactions. The skin contains twice as many T-cells as the same volume of blood as well as langerhans cells, skin-resident dendritic cells, which is usually the first tissue targeted by the host’s immune response in a vascularized composite allotransplantation (VCA) (67–70). As a result, several studies have suggested the inclusion of a skin component to monitor for early signs of JA rejection (71, 72).

Muscle is not essential in JA, but may be required in cases of extensive tissue loss. While muscles are less prone to lymphocytic infiltration and acute rejection due to their comparably low number of immune cells, they still carry a risk of atrophy and fibrosis, which may ultimately impact joint function (42, 73).

## Immunosuppressive or immuno-regulating treatment strategies

**Figure 3:**
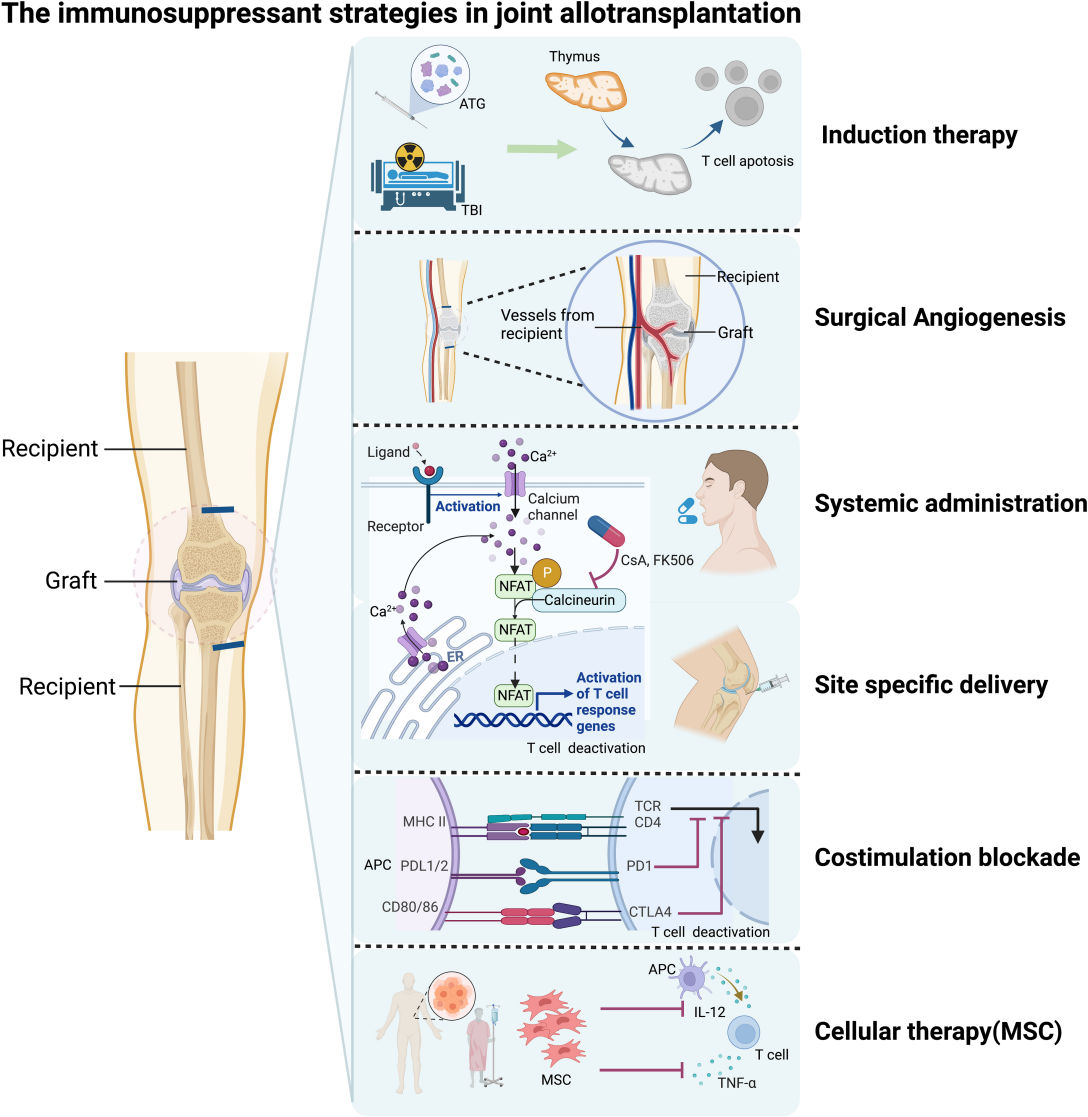
Immunosuppressive strategies for joint allotransplantation (JA). Induction therapy includes total body irradiation (in experimental settings) and anti-thymocyte globulin (ATG). Surgical neoangiogenesis can be achieved through microsurgery. Traditional systemic administration of immunosuppressive drugs is one option. Site-specific delivery of immunosuppressive drugs to the transplant site can also be used. Costimulation blockade treatment is a newer approach that targets T cell activation and proliferation. Cellular therapy, including mesenchymal stem cells (MSCs) and hematopoietic cell transplantation, is another promising strategy that has shown potential in preclinical studies. Figure created with BioRender.com.

The primary objective of immunosuppression or immunoregulation in VCA, including JA, is to prevent immune rejection or, ideally, establish long-term immune tolerance, ultimately ensuring graft survival. In general, immunosuppressive treatment strategies used in VCA, including novel approaches, can be applied to JA (**Figure 3**). A standard treatment algorithm for VCA has not been clearly defined, and the common protocols used in clinics for immunosuppression and rejection management in VCA, usually derived from solid organ transplantation which can be classified in induction, maintenance, and rescue therapy (74–78).

T-cell depleting agents such as antithymocyte immunoglobulin (ATG), basiliximab, and alemtuzumab are commonly used for immunosuppressive induction to prevent early acute rejection and graft loss (74–76). To date, ATG has been exclusively used for face transplant recipients, while all three agents are equally utilized in hand transplantation (74).

Triple therapy with calcineurin inhibitors tacrolimus, mycophenolate mofetil (MMF), and corticosteroids is typically used as maintenance immunosuppression in VCA (75, 76). Dual-steroid-free maintenance immunosuppression has also been reported and shows long-term allograft survival with infrequent and manageable complications in a series of VCA recipients (77).

Pulsed steroid therapy and increasing maintenance immunosuppression have successfully been used as rescue therapy. For steroid-resistant acute rejections, ATG and alemtuzumab are beneficial (78).

In the previously reported knee allotransplantation series, the first five cases used anti-thymocyte globulin as an induction therapy, along with cyclosporin A, azathioprine, and methylprednisolone as immunosuppressive regimens. In the sixth and last patient of the series, tacrolimus and MMF were used instead of cyclosporin A and azathioprine. This last patient showed better graft survival and functional outcomes compared to the first five patients (3). Considering the unique physiological and anatomical characteristics of JA, novel therapeutic approaches should also be discussed.

One promising treatment approach to reduce graft immunogenicity is surgical neoangiogenesis, which is distinct from whole limb transplantation. Joint allografts are interposed between recipient tissue on both ends, providing an opportunity to introduce recipient-specific blood supply and limit the size of the required donor pedicle. Previous studies have demonstrated that longer and thicker vascular pedicles from the donors are more likely to develop intimal hyperplasia, significant fibrotic perivascular tissue formation, and ischemia due to untreated episodes of acute rejection, leading to graft vasculopathy and poor survival of vascularized bone and joint allografts (4, 79). A saphenous arteriovenous (AV) bundle from the recipient has been used successfully to generate neoangiogenic bone circulation in JA (23, 48).

Another emerging approach to immunological control in VCA is site-specific delivery systems for immunosuppression. These systems have the potential to reduce the necessary systemic immunosuppression dosage, thereby alleviating its toxicity and side effects while maintaining sufficient drug levels within the graft to prevent rejection. The direct accessibility of the graft for these local interventions represents a native advantage in VCA (80). High-dose intra-graft administration of tacrolimus has been shown to prolong allograft survival, even when the drug is not detectable systemically (81). Similarly, site-specific application of tacrolimus-loaded hydrogel had a similar effect (82, 83). Other methods of site-specific immunosuppression delivery, such as implants and nanoparticles loaded with rapamycin, have also been employed successfully (84, 85). For JA, immunosuppression could potentially be delivered directly into the joint or surrounding tissue. However, at present, no such studies have been performed, and it remains to be seen whether the clinical outcomes of JA can be significantly improved by these methods.

Cellular therapies based on mesenchymal stem cells (MSCs), including bone marrow-derived MSCs and adipose-derived stem cells, have been explored as adjuvant therapy to traditional immunosuppression in allotransplantation. MSCs can increase the number of regulatory T-cells through various mechanisms, including TGF-B and indoleamine 2,3-dioxygenase, as well as decrease the expression of proinflammatory cytokines (TNF-α and IL-12) in circulation (52, 86, 87). Recent studies have demonstrated that after T cell-replete hematopoietic cell transplantation with induction therapy, VCAs rapidly become infiltrated with recipient T-cells in the absence of gross or histological evidence of rejection, resulting in cutaneous T-cell chimerism and suggesting that the infiltrating cells are rapidly tolerized (88). Because of their tissue composition, VCAs may be considered to carry an innate pool of MSCs. Whether grafted MSCs can exert a significant immunosuppressant effect in JA remains to be seen.

Costimulation blockade (CoB) has emerged as a promising approach for allotransplantation, including VCA (89). CTLA4-IgG1 or belatacept, a fusion protein of CTLA4 and IgG1, can effectively inhibit T- and B-lymphocyte activity and prevent immune rejection *via* CD28/B7 costimulation between T- and B-lymphocytes (90). A recent study in rats induced mixed chimerism and revealed intrinsic tolerogenic potential using CoB (31). Similar results have been demonstrated by other animal researches (91). Clinical case also showed belatacept can provide sufficient prophylaxis from rejection without chronic calcineurin inhibitors-associated side effects in VCA (92). However, no experimental studies have explored the impact of CoB in a JA model.

The question remains whether the benefits of functional joint reconstruction justify the potential costs of surgical failure and life-long immunosuppression side effects. These challenges considerably restrict the clinical application of JA, often leaving patients who do not qualify for an artificial joint replacement with few alternatives besides arthrodesis or amputation. However, the most recent JA clinical cases were performed over a decade ago, and ongoing clinical and preclinical trials for new immunosuppression therapies could help overcome the primary hurdles of rejection and immunosuppression-related toxicity.

Current research has demonstrated that induction therapy, such as total body irradiation and anti-thymocyte globulin combined with any of the treatments mentioned above, can prolong graft survival time and even result in chimerism and immune tolerance, particularly in small animal models. However, unlike life-threatening diseases, JA and other VCA pose long-term threats to patients. For young individuals and others, potential side effects that can result in death or disability are far more concerning than the possibility of functional restoration. Therefore, minimizing the side effects of immunosuppression is of paramount importance, given the significant potential for JA’s widespread clinical use. Promising prospects for local immunosuppression in the short term, as well as immunoregulation, exist for achieving this goal.

## Functional reconstruction

Functional reconstruction is the primary objective of JA and it is both theoretically and practically feasible to improve the quality of life through this procedure. Several studies have reported successful functional outcomes following JA, such as a study of hand transplantation which demonstrated a satisfying functional outcome over 10 postoperative years, and a knee transplantation case which achieved a range of motion almost equivalent to that of a healthy joint (3, 37, 39). However, restoring mobility and stability involves various structures, including muscles, nerves, and accessory structures around the joint, all of which are crucial to its static and dynamic function. The intrinsic function of a joint includes movement, weight-bearing, and buffering among other factors.

Achieving proper weight-bearing function after JA can be challenging, particularly as in VCA there are no direct comparisons due to the lack of leg transplant cases. Although previous studies have shown that an orthotopic load-bearing porcine forelimb VCA was successfully established within 14 days, patients who received knee transplants eventually lost their ability to fully or partially weight-bear due to complications such as fatigue fractures and infections (3, 33). Therefore, it is crucial to ensure good intraoperative bony fixation during the transplantation procedure to support weight-bearing ability. Moreover, preventing fractures in the long term is a significant concern as inflammation caused by rejection can weaken the bone and increase the risk of fractures. Hence, appropriate measures must be taken to prevent rejection and maintain the structural integrity of the transplanted joint to ensure optimal functional outcomes (93, 94). This includes adequate postoperative management, such as regular follow-up appointments to monitor bone density, physical therapy to strengthen muscles around the joint, and obviously appropriate immunosuppressive medication to prevent rejection.

Restoring motor function can be challenging and involves movement around one or more axes, depending on the specific joint. Joints act as a lever point around which the surrounding muscles generate movement (95). Each motor function typically involves the coordinated movement of entire muscle groups around the joint, rather than a single muscle (96). Reduced muscular excursion due to prolonged immobilization in patients who are candidates for JA makes restoring motor function a lengthy process that requires ongoing rehabilitation and physical training. Previous knee transplantation cases have shown improvement in the range of motion up to 1.5 years after transplantation (4).

Joints also act as buffers against movement and force, such as the meniscus in the knee joint, which plays a significant role in absorbing impact during activities like running and jumping. Preventing long-term collagen absorption and rejection in allografted menisci is challenging, and once the meniscus is affected by immune rejection, functional reconstruction of the knee becomes hard to maintain. Non-vascularized cartilage replacements including autografts and allografts have been used in the clinical setting to treat degenerative cartilage disorders. Previous studies have shown that the mean survival rate of osteochondral allografts in the patellofemoral joint was 87.9% at 5 years and 77.2% at 10 years (97). However, in JA, the cartilage, including the meniscus, is only part of a larger composite allograft and is therefore potentially more exposed to inflammation and immune rejection.

Thus, adequate immunosuppression is essential in JA to ensure long-term functional restoration and rehabilitation. Any inflammatory reaction in the graft from immune rejection will cause swelling and pain, leading to disturbances in motor function and eventually the failure of the desired functional reconstruction (98, 99). Therefore, careful management of immunosuppression is critical for maintaining long-term graft survival and improving the functional outcomes of JA.

As restoration of function is a primary goal of JA, it is crucial to have adequate assessment tools for accurate documentation of the functional reconstruction process. Kubiak et al. proposed three categories of functional assessment after VCA: surveys, observational rating of performance, and kinematic evaluations (100). These categories should be customized based on the specific joint being transplanted. In the clinical setting, several surveys and rating instruments, such as the Disabilities of the Arm, Shoulder and Hand questionnaire (DASH), are available for functional assessment. Additionally, kinematic evaluations provide objective measurements of motor function and strength (100–103). To evaluate functional parameters in animal JA models, passive range of motion, cantilever bending, and elasticity of cartilage can be assessed (48). Other investigations, including scintigraphy, sonography, angiography, electrophysiological examination, and CT-bone scans, can be used to verify the postoperative viability and perfusion of the transplanted joint (49). To objectively assess motor function in animal models, several methods such as axial compression test, Basso, Beattie and Bresnahan (BBB) locomotor scale method, and high-speed motion capture systems to measure three-dimensional kinematics of joints can be used (104–106).

## Prospects in joint allotransplantation

While prosthetic replacements offer functional restoration without immune rejection, they also have their own problems and complications (107, 108). JA can be considered as a last salvage option for patients with advanced joint destruction. Although JA offers a promising alternative to arthrodesis and amputation, its clinical applications are insignificant compared to prostheses and function-sacrificing surgical procedures. The issue of immune rejection remains a significant obstacle, and several unsolved questions continue to hinder the widespread use of this reconstructive option. However, the clinical demand for functional reconstruction in young individuals and cases that do not qualify for prosthetic solutions warrants further investigations in the field of JA. In the future, a new generation of immunosuppressive treatment options, which prolong graft survival and alleviate systemic toxicity and long-term side effects, could address these concerns and promote the clinical application of JA. Despite the limited success in past clinical cases, these emerging new immunosuppressive strategies and the persisting clinical demand warrant further investigations in the field of JA.

## Conclusion

Joint allotransplantation aims to restore joint motor function and improve the quality of life for recipients. Despite its potential benefits, research on joint-specific allotransplantation has been limited in the past two decades due to the complexity of the surgical procedures, limited clinical applications, and the risk of immune rejection, which can lead to uncertain outcomes.

To address these challenges, it is essential to prolong graft survival while minimizing the adverse effects of immunosuppressive drugs. Emerging immunosuppressive strategies offer new opportunities to improve transplantation outcomes, and further studies are needed to evaluate the feasibility and clinical potential of vascularized joint allotransplantation.

A deeper understanding of the immunological mechanisms involved in joint-specific transplantation may enable the optimization of transplantation outcomes and ultimately improve patient outcomes and quality of life. Therefore, advancing research in this field has the potential to contribute significantly to the advancement of transplantation medicine.

## Author contributions

The study was conceived and designed by RO, while LZ was responsible for data summarization and manuscript drafting. The manuscript was then revised by IH, CZ, RR, MC, and RO, who also provided important reagents and approved the final version. All authors contributed to the article and approved the submitted version. 
